# RNAi-Mediated Silencing of vATPase Subunit E Impairs Larval Development in *Plutella xylostella*, and Virtual Screening Identifies a Potential Inhibitor

**DOI:** 10.3390/insects17040439

**Published:** 2026-04-20

**Authors:** Xuetao Yu, Jinhua Luo, Jiayi Xue, Lin Lu, Pan Deng, Li Zhu, Kang Yang, Xia Wan, Yuhua Wu, Akmal Boboev, Gang Wu, Xiaohong Yan, Chenhui Shen

**Affiliations:** 1 Key Laboratory of Agricultural Genetically Modified Organisms Traceability, Yangluo Station of Chinese Agrosystem Long-Term Observation Network, Oil Crops Research Institute of Chinese Academy of Agricultural Sciences, Ministry of Agriculture and Rural Affairs, Wuhan 430062, China; xuetao.yu@outlook.com (X.Y.); 821012510043@caas.cn (J.X.); ll15966192116@163.com (L.L.); zhuli49751@163.com (L.Z.); yawa521@gmail.com (K.Y.); wanxia@oilcrops.cn (X.W.); wuyuhua@oilcrops.cn (Y.W.); wugang@caas.cn (G.W.); 2 Enshi Tujia and Miao Autonomous Prefecture Academy of Agricultural Sciences, Enshi 445000, China; chw63@126.com; 3 Institute of Leisure Agriculture, Jiangsu Academy of Agricultural Sciences, Nanjing 210014, China; shinebee3@hotmail.com; 4 Enology & Technology of Fermentation Products, Tashkent Institute of Chemical Technology, 32, Navoi Str, Tashkent 100011, Uzbekistan; akbob16@gmail.com

**Keywords:** *Plutella xylostella*, RNA interference, microinjection, virtual screening, *vATPaseE*

## Abstract

The diamondback moth (*Plutella xylostella*) is a devastating global pest of cruciferous crops. This study explores the potential of targeting the vacuolar ATPase subunit E (*PxvATPaseE*) for its control. We demonstrate that *PxvATPaseE* is essential for larval development, showing high expression levels in the midgut. RNA interference (RNAi)-mediated silencing of *PxvATPaseE* resulted in severe growth retardation and dose-dependent mortality, with higher dsRNA doses inducing more sustained effects. Furthermore, computational virtual screening of natural compound libraries identified a high-affinity binder of *Px*vATPaseE, such as periplocoside D. Our results demonstrate that *PxvATPaseE* is a promising molecular target for controlling *P. xylostella*, supporting a dual-strategy approach combining RNAi and targeted chemical inhibition for future pest management solutions.

## Simple Summary

*Plutella xylostella* poses a significant global threat to cruciferous crops such as cabbage and broccoli. Managing the pest has become increasingly difficult due to its resistance to conventional insecticides. This study identifies *PxvATPaseE*, an essential gene for larval survival and development in *P. xylostella*. Utilizing RNA interference (RNAi), we successfully reduced the mRNA level of *PxvATPaseE*, resulting in high larval mortality and severe developmental impairment. Furthermore, we employed computational virtual screening to identify natural compounds capable of inhibiting *Px*vATPaseE. These findings validate *PxvATPaseE* as a promising target for developing eco-friendly pest management methods against *P. xylostella*.

## 1. Introduction

The diamondback moth, *Plutella xylostella*, is a significant lepidopteran pest of cruciferous crops, causing major global damage, with annual economic losses estimated at US$4–5 billion [1,2]. The rapid evolution of insecticide resistance has rendered chemical control increasingly challenging. Field control still relies primarily on chemical insecticides, such as chlorantraniliprole and chlorpyrifos [2,3]. Consequently, identifying effective alternatives for managing *P. xylostella* is urgent.

RNA interference (RNAi) is a cellular process that employs double-stranded RNA (dsRNA) to suppress gene expression in a precise, sequence-specific manner, offering substantial promise for pest management [4,5,6,7,8]. Recent studies have indicated that vATPases provide a strategic entry point for RNAi-based pest management. RNAi targeting V-ATPase subunits consistently induces lethal phenotypes, and advances in dsRNA design, nanoparticle carriers, and plant-based, microbial, or spray-based delivery systems have expanded the practical application, highlighting insect ATPases as both fundamental drivers of biological processes and promising molecular targets [9,10].

The potential of RNAi has been widely demonstrated across various insect orders, including coleopterans such as *Tribolium castaneum* [11], *Leptinotarsa decemlineata* [6,12], and *Henosepilachna vigintioctopunctata* [13,14,15,16], in thysanopteran *Frankliniella occidentalis* [17], in hemipterans *Apolygus lucorum* [18] and *Nilaparvata lugens* [19], and in lepidopterans *Manduca sexta* [20] and *Helicoverpa armigera* [21]. Recent evidence demonstrates that RNAi targeting *PxPiwi* [22] or *PxTH* [23] in *P. xylostella* impairs larval development, suggesting the feasibility of RNAi-based management for this pest.

The vacuolar-type H^+^-ATPase (vATPase) is an evolutionarily conserved, multi-subunit complex in eukaryotes that hydrolyzes adenosine triphosphate (ATP) to pump protons across membranes, thereby regulating various cellular and extracellular biological processes [24,25,26]. Structurally, the V-ATPase comprises two main subcomplexes: the V_1_ complex, containing eight subunits (A–H) responsible for ATP hydrolysis, and the V_0_ complex, which facilitates transmembrane proton movement [27,28,29,30,31]. In *Tuta absoluta*, feeding on tomato leaves treated with ds*TavATPaseA* resulted in substantial larval mortality and pupation failure [32]. Both injection of dsRNA and ingestion of bacterially expressed dsRNA significantly downregulated expression of *vATPaseA* in *Hyphantria cunea*, causing high larval mortality and pupal malformation [33]. Studies on *Bemisia tabaci* have suggested that RNAi targeting different vATPase subunits may elicit distinct phenotypic effects, with silencing efficacy exhibiting biotype-specific variation [34]. In the coleopteran *Plagiodera versicolora*, ingestion of dsRNA targeting *vATPaseA* and *vATPaseE* severely suppressed development from the first to third instar and impaired adult mating or oviposition [35].

Previous studies have highlighted RNAi of the V-ATPase E subunit (vATPaseE) as a promising approach for insect pest control. For example, knockdown of *vATPaseE* causes high mortality in hemipterans like *A. lucorum* [18] and *Acyrthosiphon pisum* [20]; in coleopterans such as *T. castaneum* [11], *L. decemlineata* [36], *H. vigintioctopunctata* [14,26], and *Diabrotica virgifera virgifera* [37]; and in the lepidopteran *M. sexta* [18] and the dipteran *Drosophila melanogaster* [20]. Furthermore, expression of *vATPaseE* dsRNA in transgenic sugarcane leads to significant mortality in *Sphenophorus levis* [38]. These findings suggest that targeting *PxvATPaseE* via RNAi could be an effective strategy for controlling *P. xylostella*.

Advances in computational capabilities have greatly facilitated the screening of target protein inhibitors from large compound libraries [39,40,41]. For instance, Gryniukova et al. preselected 434 compounds for sirtuin-1 inhibition from a library of 2.6 million compounds using virtual screening [39], and Chafer-Dolz et al. discovered novel inhibitors of acetylcholinesterase through virtual screening [41]. These findings confirm that virtual screening can efficiently identify candidate inhibitors for target proteins from extensive databases.

In this study, we selected *PxvATPaseE* as a target gene to investigate the effects of RNAi on *P. xylostella*. Our approach involved (i) identifying and conducting a phylogenetic analysis of *PxvATPaseE*; (ii) characterizing its expression profiles across various developmental stages and tissues; (iii) quantifying the impact of in vitro-synthesized dsRNA on *PxvATPaseE* expression; (iv) evaluating the effects of two doses of ds*PxvATPaseE* on larval weight, survival, pupation, and adult emergence; and (v) using virtual screening to identify potential small-molecule inhibitors of *PxvATPaseE* from a library of 15,216 natural compounds.

## 2. Materials and Methods

### 2.1. Insect Rearing

*P. xylostella* larvae were collected from *Brassica napus* in Haidong City, Qinghai Province, China. They were maintained on fresh *Brassica* leaves under controlled conditions: 26 ± 1 °C, a 16:8 h (light:dark) photoperiod, and 60 ± 5% relative humidity. Adults were provided with a 10% honey solution.

### 2.2. Molecular Cloning and Phylogenetic Analysis

The sequence of *PxvATPaseE* was obtained from the genome and transcriptome data of *P. xylostella* [42,43]. The accuracy was confirmed by polymerase chain reaction (PCR) using the primers listed in Table S1. The cDNA sequence was downloaded from NCBI (GenBank accession number: AB189032.1). Phylogenetic analysis of *PxvATPaseE* was conducted using MEGA-5 software (https://sourceforge.net/projects/mega5/, accessed on 18 April 2025) and the neighbor-joining method, with 1000 bootstrap replications.

### 2.3. Synthesis of dsRNA

We designed and synthesized dsRNA targeting *PxvATPaseE* (ds*VAE*) and a control targeting the green fluorescent protein gene (ds*GFP*). cDNA fragments for both targets were PCR-amplified using specific primers flanked by T7 promoter sequences (Table S1 and Figure S1). dsRNA was synthesized by in vitro transcription using the T7 RiboMAX™ Express RNAi System (Promega, Madison, WI, USA) following the manufacturer’s protocol. The transcription reaction, containing 1 μg DNA template, 10 μL of RiboMAX™ Express T7 2× Buffer (Promega, Madison, WI, USA), and 2 μL of T7 Enzyme Mix (Promega, Madison, WI, USA), was incubated at 37 °C for 2–6 h, heat-inactivated at 70 °C for 10 min, and then slowly cooled to room temperature to facilitate dsRNA formation. The product was treated with DNase I and RNase A to remove template DNA and single-stranded RNA, respectively, and then purified using a gel extraction kit (Omega, Norcross, GA, USA). The dsRNA concentration was determined by measuring absorbance at 260 nm with a Nanodrop 1000 spectrophotometer, and its integrity was confirmed by agarose gel electrophoresis. Purified dsRNA was aliquoted and stored at −80 °C until use.

### 2.4. dsRNA Injection and Sample Collection

Microinjection of dsRNA was performed as previously described [44,45,46]. Newly molted fourth-instar larvae were selected for injection. Borosilicate glass capillaries were prepared using a PC-10 puller (Narishige, Tokyo, Japan). Using the microinjection system (World Precision Instruments, Sarasota, FL, USA), a volume of 0.2 μL containing either 800 ng or 1200 ng of dsRNA (total dose, as per reference [47]) was delivered into the larval hemolymph. Larvae injected with ds*GFP* served as the negative control. For each dsRNA treatment, six experimental replicates were performed, with each replicate consisting of eight injected larvae. Following microinjection, the larvae were individually placed in plastic boxes and supplied with fresh rapeseed leaves. To evaluate RNAi efficiency, three replicates per group were collected at 2 and 3 days post-injection for qRT-PCR analysis. The remaining three replicates were observed for three weeks to monitor phenotypic defects, larval weight change, survival, pupation, and adult emergence rates.

### 2.5. RNA Extraction and Quantitative Real-Time PCR (qRT-PCR)

To analyze temporal and tissue-specific expression patterns as well as treatment effects, RNA was isolated from the collected samples. Specifically, total RNA was extracted from treated larvae (eight individuals pooled per sample) using the Total RNA Extraction Reagent (YiFeiXue Tech, Nanjing, China), with three independent biological replicates prepared for each condition. Quantitative real-time PCR (qRT-PCR) was performed following a previously established method [48] to quantify transcript levels. *RPL32* was used as the reference gene for normalization. Each biological replicate was assayed in three technical replicates. Relative mRNA expression was calculated using the 2^−ΔΔCT^ method.

### 2.6. Virtual Screening for PxvATPaseE Inhibitors

The three-dimensional structure of the *Px*vATPaseE protein was predicted using AlphaFold 3 [49], an artificial intelligence-based modeling tool. A comprehensive compound library was constructed by integrating multiple chemical databases. A total of 15,216 natural compounds were obtained after preprocessing, which included conversion from 2D to 3D structures, addition of hydrogen atoms, assignment of Gasteiger charges, energy minimization, and application of the MMFF94 force field.

Hierarchical virtual screening was then performed using the molecular docking program AutoDock Vina (version 1.2.5) [50], a standard physics-based docking tool. First, high-throughput virtual screening (HTVS) was conducted. Compounds prone to computational errors due to unreasonable bonds, heavy atoms, excessive cavities, or high torsional degrees of freedom were removed, resulting in valid docking scores for 14,468 compounds. The top 10% of compounds ranked by “Max Affinity” from the HTVS were selected for the second round of virtual screening. Subsequently, the top 5% of compounds from this screening round (based on “Max Affinity”) were subjected to a final refined screening step to obtain the ultimate “Max Affinity” rankings. The modeled structure of *Px*vATPaseE was based on docking by AutoDock Vina. The structural data were processed using the PyMOL software (version 2.5.5). This work was performed in collaboration with Hefei Kejing Biotechnology Co., Ltd. (Hefei, China).

### 2.7. Statistical Analysis

Statistical analyses were conducted using SPSS (version 29.0.2.0) for Windows (Chicago, IL, USA). Mean differences (±SE) were analyzed by one-way ANOVA with the Tukey–Kramer post hoc test. Survival curves were analyzed with the log-rank test (Mantel–Cox; 95% CI) in GraphPad Prism (version 8.0).

## 3. Results

### 3.1. Identification and Phylogenetic Analysis of PxvATPaseE

The putative full-length *PxvATPaseE* was confirmed in *P. xylostella* by mining the transcriptome data (Figure S1). The *PxvATPaseE* consisted of a 681 bp complete open reading frame, encoding 226 amino acid residues (Figure S1). The calculated molecular weight and isoelectric point of *PxvATPaseE* were 26.13 kDa and 7.78, respectively, as determined by the Compute pI/MW tool (https://web.expasy.org/compute_pi/, accessed on 18 April 2025).

**Figure 1:**
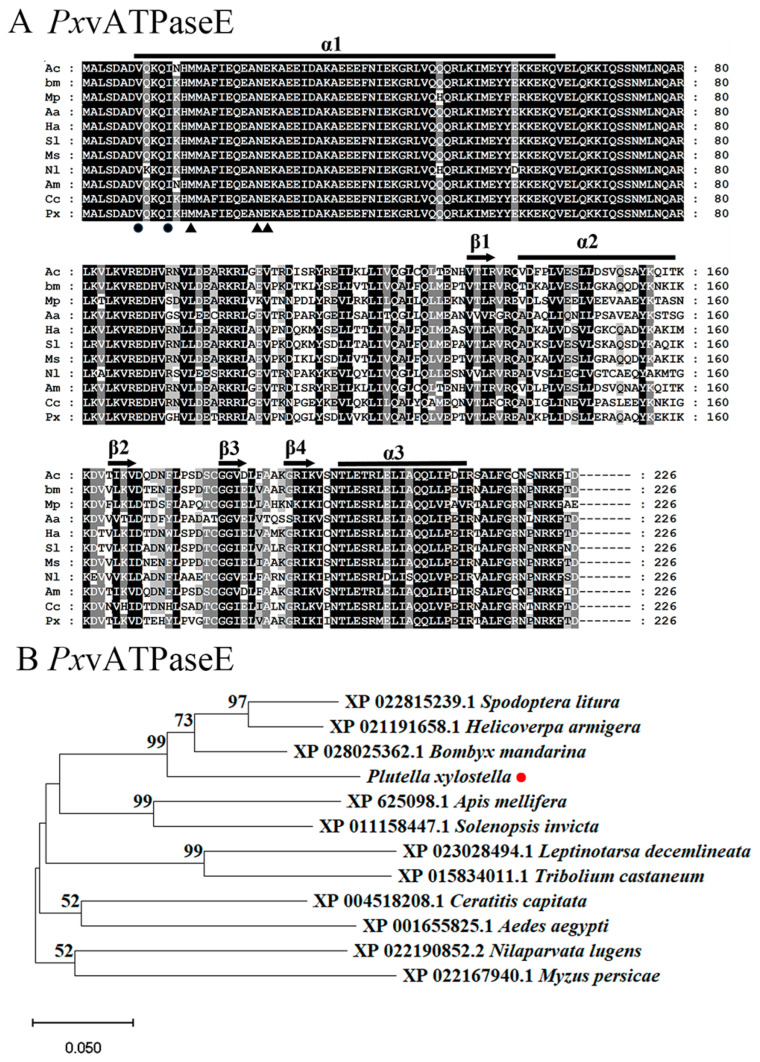
**Sequence alignment and phylogenetic analysis of V-ATPase subunit E from *P. xylostella*.** (**A**) Multiple sequence alignment of vATPaseE proteins from *Apis cerana* (Ac), *Bombyx mandarina* (Bm), *Myzus persicae* (Mp), *Aedes aegypti* (Aa), *Helicoverpa armigera* (Ha), *Spodoptera litura* (Sl), *Manduca sexta* (Ms), *Nilaparvata lugens* (Nl), *Apis mellifera* (Am), *Ceratitis capitata* (Cc), and *Plutella xylostella* (Px). The background shading intensity is proportional to sequence similarity (light to dark). Gaps are introduced for optimal alignment. Predicted secondary structure elements (α-helices and β-sheets) of subunit E, arranged in order α1:β1:α2:β2:β3:β4:α3, are indicated above the alignment (lines for α-helices; arrows for β-sheets). Triangles and circles mark amino acid residues critical for interaction with subunits G and C, respectively. (**B**) Phylogenetic tree of *vATPaseE* proteins from four lepidopteran (*Spodoptera litura*, *Helicoverpa armigera*, *Plutella xylostella*, and *Bombyx mandarina*), two hymenopteran (*Apis mellifera* and *Solenopsis invicta*), two dipteran (*Aedes aegypti* and *Ceratitis capitata*), two hemipteran (*Nilaparvata lugens* and *Myzus persicae*), and two coleopteran (*Leptinotarsa decemlineata* and *Tribolium castaneum*) species. The tree was constructed using the neighbor-joining method based on full-length protein sequence alignments. Bootstrap support values from 1000 replicates are shown at nodes (>50%).

A sequence alignment revealed that vATPaseE proteins in different insects were highly conserved. The vATPaseE protein consisted of four *β*-strands (S131-I134, K164-V167, G179-E182, and G188-K191) and two *α*-helices (E139-L159 and T195-I211). The sheets and helices were connected by flexible loop regions in the following order: β1:α2:β2:β3:β4:α3 (Figure 1A).

To elucidate the evolutionary relationships of vATPaseE-like proteins, a phylogenetic tree was constructed using sequences from 12 species (Figure 1B). The analysis included species from five insect orders: Lepidoptera (*P. xylostella*, *Bombyx mandarina*, *H. armigera*, and *Spodoptera litura*), Hymenoptera (*Apis mellifera* and *Solenopsis invicta*), Diptera (*Aedes aegypti* and *Ceratitis capitata*), Hemiptera (*N. lugens* and *Myzus persicae*), and Coleoptera (*T. castaneum* and *L. decemlineata*). The resulting unrooted tree demonstrated that vATPaseE-like proteins from species within the same order clustered together. Accordingly, vATPaseE from *P. xylostella* was clearly grouped within the lepidopteran clade (Figure 1B).

### 3.2. Expression Profiles of PxvATPaseE

**Figure 2:**
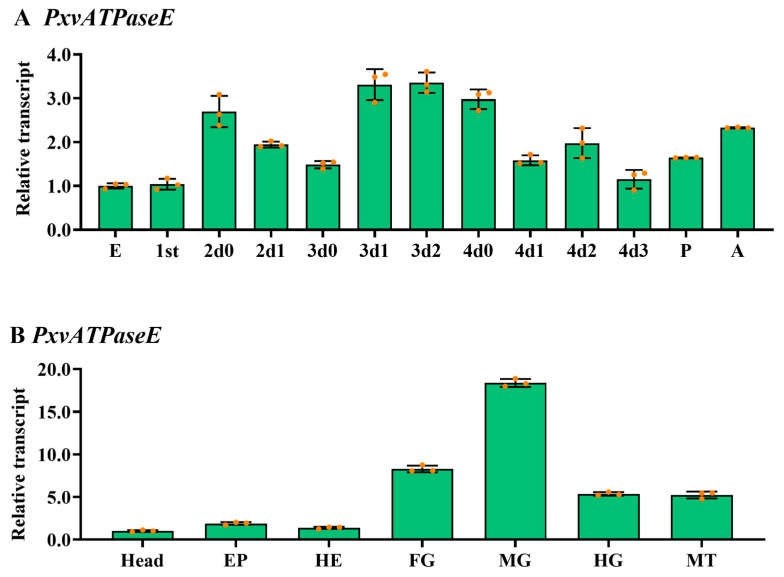
**Temporal and spatial expression profiles of *PxvATPaseE* in *P. xylostella*.** (**A**) Temporal expression. Complementary DNA templates were prepared from eggs; larvae of the first (0–1 day post-hatching), second, third, and fourth instars (day 0 indicates newly molted individuals); newly ecdysed pupae; and newly emerged adults. (**B**) Tissue-specific expression. Templates were derived from the head, foregut (FG), midgut (MG), hindgut (HG), hemolymph (HE), Malpighian tubules (MT), and epidermis (EP) of day-4 fourth-instar larvae. Expression levels were calculated using the 2^−ΔΔCT^ method, normalized to the lowest level (set as 1) observed in eggs and head tissues. Data are presented as means ± standard errors (SEs).

The temporal expression pattern of *PxvATPaseE* was examined by qRT-PCR. Transcripts were detectable across all developmental stages from embryo to adult. Expression peaked on day 2 of the third-instar larval stage, while the lowest level was observed at the egg stage. A consistent expression pattern was noted throughout larval development: mRNA levels increased during each ecdysis period and decreased during the subsequent feeding stage (Figure 2A). Spatial expression profiling was performed in multiple tissues of fourth-instar larvae, including the head, foregut, midgut, hindgut, hemolymph, Malpighian tubules, and epidermis. *PxvATPaseE* expression was highest in the midgut and foregut, moderate in the hindgut and Malpighian tubules, and low in the head, hemolymph, and epidermis (Figure 2B).

### 3.3. RNAi of PxvATPaseE in Fourth-Instar P. xylostella Larvae (800 ng Dose)

**Figure 3:**
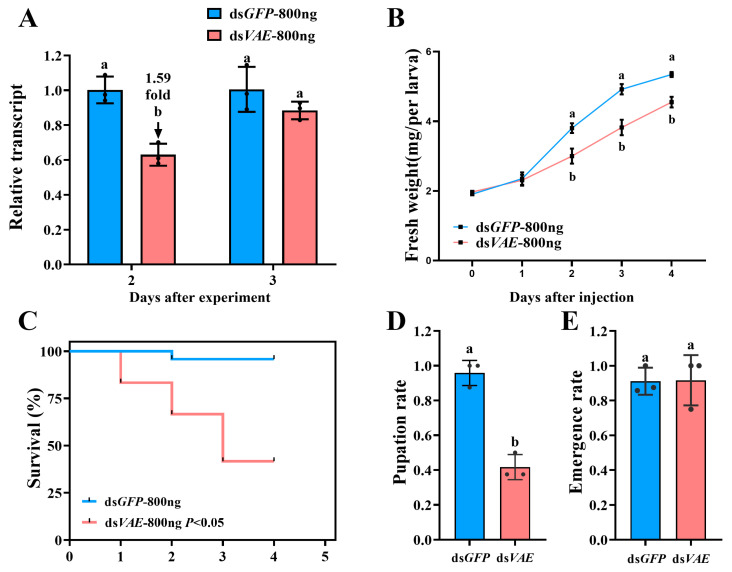
**Effects of *PxvATPaseE* silencing in fourth-instar larvae of *P. xylostella*.** Newly molted fourth-instar larvae were injected with 0.2 µL of a solution containing 800 ng of ds*VAE*; ds*GFP*-injected larvae served as a negative control. After injection, the larvae were reared on fresh rape leaves. (**A**) Relative *PxvATPaseE* expression levels at 48 and 72 h post-injection, normalized to ds*GFP* control (set as 1). (**B**–**E**) Larval fresh weight (**B**), survival rate (**C**), pupation rate (**D**), and adult emergence rate (**E**) were monitored over a 5-day period. Data are presented as means ± SEs. Different letters indicate statistically significant differences (*p* < 0.05).

To evaluate the effects of RNAi targeting *PxvATPaseE* in *P. xylostella*, we silenced the gene via dsRNA injection. Delivery of 800 ng of ds*VAE* into newly molted fourth-instar larvae significantly reduced *PxvATPaseE* mRNA levels by approximately 1.59-fold at day 2 post-injection, although no significant reduction was observed at day 3 (Figure 3A).

**Figure 4:**
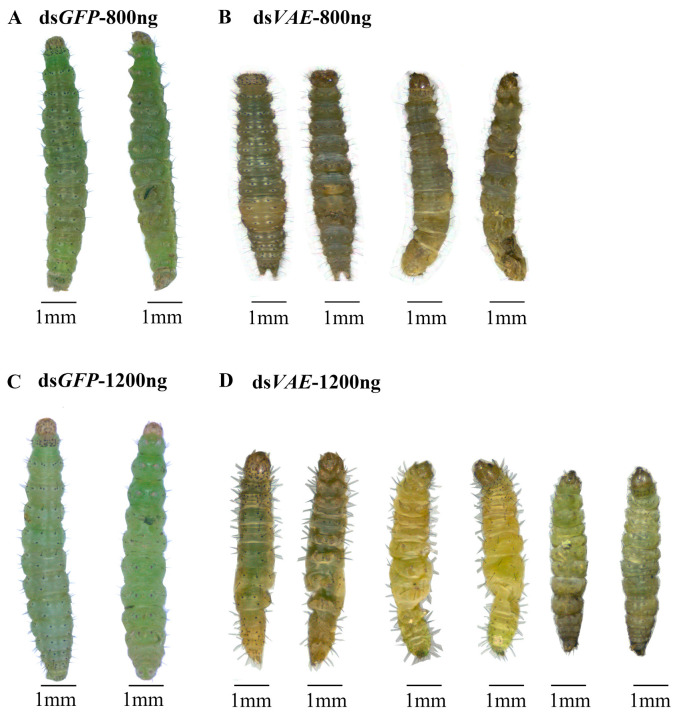
**Phenotypic consequences of *PxvATPaseE* knockdown in *P. xylostella* fourth-instar larvae.** Larvae were injected with ds*VAE* (800 ng or 1200 ng) or an equivalent amount of ds*GFP* (control) and reared on rape leaves. Developmental progression was monitored at 24 h intervals. Representative phenotypes of ds*VAE*-treated and control larvae were documented on day 3 post-injection.

Knockdown of *PxvATPaseE* markedly suppressed larval development, resulting in fresh weight reductions of 19.9%, 22.2%, and 14.8% at days 2, 3, and 4, respectively, compared with ds*GFP*-injected controls (Figure 3B). Furthermore, *PxvATPaseE* silencing caused substantial larval mortality, reaching 16.7%, 33.3%, and 58.3% at days 2, 3, and 4 after treatment, respectively (Figure 3C). Of the surviving larvae, 41.7% proceeded to pupation (Figure 3D), while no significant difference in adult emergence rate was detected between ds*VAE*- and ds*GFP*-treated groups (Figure 3E). Notably, ds*VAE*-treated larvae exhibited malformed and smaller bodies, followed by gradual dehydration, darkening, and eventual death (Figure 4A vs. Figure 4B).

### 3.4. Enhanced Effects of a Higher dsVAE Dose (1200 ng)

**Figure 5:**
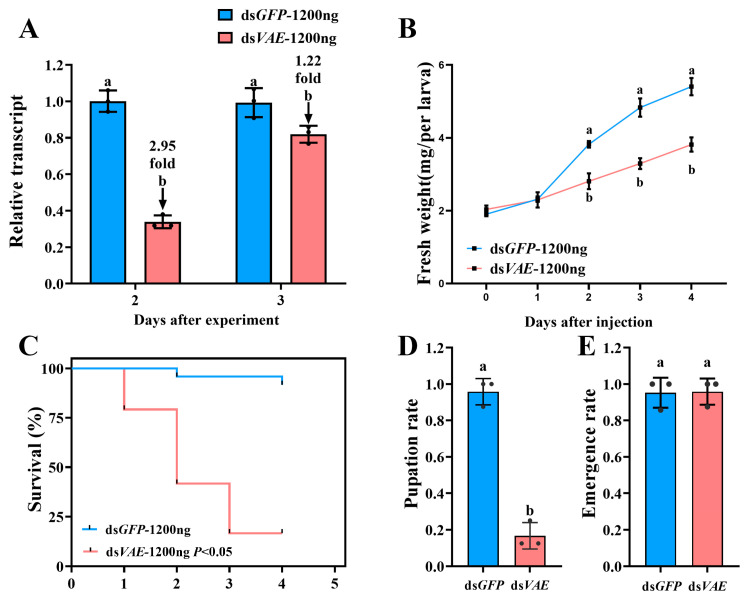
**Effects of *PxvATPaseE* knockdown in *P. xylostella* fourth-instar larvae (1200 ng of dsRNA dose).** Newly molted fourth-instar larvae were injected with 0.2 µL of a solution containing 1200 ng of ds*VAE*; ds*GFP*-injected larvae served as a negative control. (**A**) Relative *PxvATPaseE* expression levels. (**B**) Larval survival rate. (**C**) Larval mortality rate. (**D**) Pupation rate. (**E**) Adult emergence rate. Data are presented as means ± SEs. Different letters indicate statistically significant differences (*p* < 0.05).

At a higher dose of 1200 ng of ds*VAE*, RNAi efficacy was enhanced, with a longer duration of action. *PxvATPaseE* mRNA levels were reduced by approximately 2.95-fold at day 2 and 1.22-fold at day 3 (Figure 5A). Larval growth inhibition was more pronounced, with fresh weight reductions of 26.5%, 31.8%, and 29.4% on days 2, 3, and 4, respectively (Figure 5B). Mortality rates increased dose-dependently, reaching 20.8%, 58.3%, and 83.3% at the same time points (Figure 5C), and only 16.7% of larvae successfully pupated (Figure 5D). As with the lower dose, the adult emergence rate did not differ significantly from the control group (Figure 5E). Phenotypic defects were more severe: many larvae failed to molt into pupae, and 83.3% of stunted larvae exhibited progressive withering, desiccation, and darkening before death (Figure 4C,D).

### 3.5. Virtual Screening Identifies Periplocoside D as an Effective Binder of PxvATPaseE

**Figure 6:**
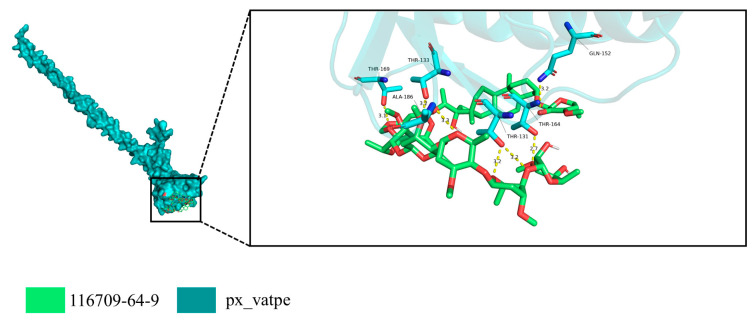
**Predicted binding mode of periplocoside D in the V-ATPase subunit E pocket.** The overall structure of PxvATPaseE is shown in cyan, with the binding pocket location indicated by a black box (left panel). An enlarged view of the pocket (right panel) shows the overlay of periplocoside D (compound 116709-64-9, green) within the same site. The two molecules share high structural overlap, suggesting similar binding modes. Key interacting residues (e.g., THR-131, THR-133, THR-164, THR-169, GLN-152, and ALA-186) form multiple hydrogen bonds or polar contacts with the ligand, as indicated by yellow dashed lines (bond distances: 2.7–3.4 Å, within the conventional hydrogen-bond distance range).

We performed virtual screening of a multi-library collection of 15,216 natural compounds using AutoDock Vina to identify high-affinity binders for the bait protein *Px*vATPaseE. The top 34 compounds with the highest predicted binding affinities are listed in Table 1. Notably, previous studies showed periplocoside D (116709-64-9) was lethal to *P. xylostella*, with an LC50 of 1310 μg/mL [51]. Our docking analysis revealed that periplocoside D might bind to *Px*vATPaseE, indicating its potential inhibitory effect on *P. xylostella* via targeting *Px*vATPaseE, thus qualifying it as a candidate compound. Finally, competitive binding within the same protein-binding pocket and their interactions with key surrounding amino acid residues were analyzed (Figure 6). Overall, Figure 6 demonstrates that both molecules compete for the same pocket and form similar interaction networks, where hydrogen bonds mediated by multiple threonine and glutamine residues constitute the primary binding force. Variations in the orientation of terminal substituents could modulate binding strength and molecular selectivity.

## 4. Discussion

In this study, we identified and characterized the V-ATPase subunit E gene (*PxvATPaseE*) in *P*. *xylostella*. Functional analysis via RNA interference (RNAi) demonstrated that silencing *PxvATPaseE* severely impaired larval survival and development. Furthermore, by combining computational virtual screening, we identified a series of natural compounds capable of binding to *Px*vATPaseE and simulated the interaction mechanism of periplocoside D with this target, thereby proposing a novel strategy for screening potential insecticidal compounds.

### 4.1. RNAi-Based Pest Control: Feasibility and Target Selection

RNAi has emerged as a potent and species-specific strategy for next-generation pest management [4,7,52,53,54]. Its application progresses along two primary pathways: the development of exogenous RNAi biopesticides [10] and the creation of transgenic crops expressing RNAi constructs. The latter approach has successfully controlled pests including *A. lucorum* [18], *L. decemlineata* [55], *Adelphocoris suturalis* [56], *Sitobion avenae* [57], and *M. persicae* [58]. Furthermore, integrating RNAi with conventional tactics synergistically overcomes pest resistance and enhances control efficacy. For example, co-expressing *hpPXCHS1* with the Bt protein in oilseed rape potentiates Bt toxicity [1], while silencing the immune-related gene *Pxdorsal* increases *P. xylostella* larval susceptibility to Bt through cuticular melanization [59]. Collectively, these advancements underscore the viability and promise of RNAi-based crop protection strategies. The success of RNAi hinges on selecting essential target genes whose disruption induces lethal or sublethal phenotypes. Effective targets are often associated with fundamental physiological pathways, including core cellular functions (e.g., vacuolar ATPase [60]), energy metabolism, and critical signaling networks governing ecdysis [11,61,62,63,64,65,66]. The demonstrated efficacy against genes such as *PxPiwi* and *PxTH* in *P. xylostella* confirms this approach’s technical feasibility [22,23]. Our work extends this principle by systematically evaluating *PxvATPaseE*, a gene encoding the core component of an evolutionarily conserved proton pump, as a novel target.

### 4.2. PxvATPaseE as a Potential Target Gene for Controlling P. xylostella Larvae

Our comprehensive analysis provides strong evidence that *PxvATPaseE* is a high-value candidate gene for RNAi-mediated control of *P. xylostella*. This conclusion is supported by multiple lines of evidence.

First, the evolutionary conservation of *vATPaseE* (Figure 1) suggests its non-redundant, essential role in cellular physiology across insects [26,28,67]. This conservation often translates to robust and predictable RNAi outcomes across species.

Second, the spatiotemporal expression profile of *PxvATPaseE* aligns with its putative function and vulnerability. Its peak expression during the third instar (Figure 2) coincides with a period of rapid growth and high metabolic demand. Most notably, its abundant transcription in the midgut (Figure 2) underscores a critical role in gut function, specifically in nutrient absorption and ion homeostasis as documented in other insects [34,68,69]. Disrupting a gene highly active in a vital tissue like the midgut provides a rational explanation for the severe growth inhibition and mortality observed post-RNAi.

Third, our functional validation yielded unequivocal results. RNAi-mediated knockdown of *PxvATPaseE* caused dose-dependent larval mortality, significant growth retardation, and failed pupation (Figure 3 and Figure 5). The phenotypic progression—body malformation, wilting, and darkening—is consistent with systemic physiological collapse, primarily driven by disruption of cellular pH homeostasis and V-ATPase-dependent energy metabolism. The efficacy of targeting *vATPaseE* is well-established; high mortality has been reported following its silencing in pests like *A. lucorum*, *L. decemlineata*, and *H. vigintioctopunctata* [18,24,26]. Our findings confirm that *P. xylostella* is equally susceptible to this approach. The clear dose–response relationship, where 1200 ng of dsRNA induced stronger and more prolonged gene suppression along with higher mortality relative to 800 ng (Figure 3 and Figure 5), highlights the importance of optimizing delivery parameters for practical application. This dose-dependent efficacy aligns with core principles of RNAi in insects [5,70] and is observed across diverse species [71,72,73].

Although RNAi was originally considered highly specific, off-target effects remain a concern. Therefore, rigorous validation is essential to ensure that dsRNA design minimizes potential impacts on non-target organisms, crops, or mammals. Moreover, silencing genes involved in regulatory functions or various metabolic pathways, such as transcription factors or signaling molecules, may lead to unintended effects in the host. Thus, multi-level off-target evaluation is critical. First, dsRNA should exclusively target the intended mRNA without affecting endogenous genes. Second, off-target risks must be evaluated in phylogenetically related insects, particularly beneficial species. Selecting appropriate target genes through screening is a suitable approach to ensure that non-pest insects are not harmed.

In this study, we employed the online tool DSRNA-Engineer (https://www.dsrna-engineer.cn, accessed on 18 April 2025) to predict the number and positions of on-target and off-target sites for the *PxvATPaseE* gene fragment during RNAi [74]. The blue curve represents on-target sites, while the red curve indicates off-target sites (Figure S2). Predictions were performed primarily for arthropod species with predatory relationships or evolutionary similarity to the target organism, aiming to identify potential off-target regions in non-target organisms. We deliberately selected regions with low probability of off-target effects as templates for dsRNA synthesis, thereby minimizing such effects.

Currently, off-target prediction relies on bioinformatics approaches using existing gene sequence databases. Therefore, developing comprehensive whole-genome and transcriptome databases for non-pest insect species is essential to predict possible off-target effects [75].

### 4.3. Nanomaterial-Assisted Efficient Delivery of dsRNA for Pest Management

Several factors influence RNAi efficiency, with dsRNA stability being a critical determinant. Once dsRNA enters the insect body, the exposed dsRNA is susceptible to degradation by nucleases present in the saliva, hemolymph, and gut, which partially reduces RNAi efficiency in certain insect species. In lepidopteran insects specifically, nucleases termed RNAi efficiency-related nucleases (REases) have been identified [76]. Additionally, studies have shown that insect hemolymph and gut fluids contain double-stranded ribonucleases (dsRNases), considered major factors limiting RNAi efficiency in oral delivery due to their impact on dsRNA stability in body fluids [77,78]. Encapsulating dsRNA with protective nanomaterials can effectively preserve its integrity, and reducing dsRNase activity helps minimize dsRNA degradation within cells, thereby enhancing RNAi efficiency. This represents a novel delivery strategy for RNAi-based applications. These nanocarrier complexes facilitate cellular uptake of dsRNA due to their high transduction efficiency and low cytotoxicity, while reducing degradation risks by nucleases under environmental conditions such as temperature fluctuations and medium changes [79].

The most extensively studied nanocarriers include chitosan, liposomes, star-shaped cationic polymers (SPcs), layered double hydroxides (LDHs), and guanidinylated polymers (GNPs). RNAi efficiency in lepidopteran insects is relatively low. A study reported the development of a chitosan-mediated oral dsRNA delivery method in silkworm larvae for the first time, which successfully induced significant knockdown of various immune gene transcripts. Prior RNAi studies in the lepidopteran model insect *Bombyx mori* were primarily conducted via injection. This advancement facilitates the application of RNAi for pest control in lepidopteran species [80]. Spray-Induced and Nanocarrier-Delivered Gene Silencing (SI-NDGS) technology, utilizing star-shaped polymer (SPc) nanoparticles, was employed to evaluate the control efficacy of different vATPase subunits in *Sogatella furcifera*. This approach successfully reduced target mRNA levels and vATPase enzyme activity while assessing the environmental safety of nanoparticle-encapsulated dsRNA [81]. In *Earias vittella*, three different nanoparticle-encapsulated dsRNA complexes significantly knocked down multiple genes, including *vATPase* [82]. For controlling the pest *H. armigera*, ionotropically synthesized cationic chitosan nanoparticles (CNPs) demonstrated high dsRNA loading efficiency and effectively protected dsRNA from degradation by gut nucleases and pH fluctuations in the insect gut [83]. Lipoplexes exhibited excellent protection of dsRNA and siRNA against heat, UV radiation, and RNase degradation, effectively suppressing gene expression, impairing larval development, and causing high mortality in early instar larvae [84]. Loading dsRNA targeting the *vATPaseA* gene onto layered double hydroxide (LDH) enhanced its environmental stability, leading to mortality in *Holotrichia parallela* larvae and disrupting their cuticle and midgut structure [85].

### 4.4. Virtual Screening Identifies a Putative PxvATPaseE Inhibitor

Beyond genetic suppression, we explored the potential for chemical inhibition of *Px*vATPaseE. The V-ATPase complex is a validated target for insecticide development [86], as demonstrated by the activity of vanillin derivatives against *Mythimna separata* [74] and isoxazoline compounds against *P. xylostella* [87]. Building on this, we employed structure-based virtual screening to identify specific inhibitors of the vATPaseE subunit. The application of computational virtual screening significantly accelerates the mining of large chemical libraries by prioritizing candidates for experimental validation [39,40,41]. Our screen of over 15,000 natural compounds identified periplocoside D among the top hits (Table 1). This finding is particularly significant in light of prior evidence: periplocoside D is known to be lethal to *P. xylostella* [51]. Previous studies have identified six periplocosides from *Periploca sepium Bunge*. Among these, compounds PSD (periplocoside D) and PSF (periplocoside F) exhibited insecticidal activity against *M*. *separata* and *P*. *xylostella*, with 48 h median lethal dose (LD50) values of 12.17 and 13.95 μg per larva [88]. Notably, its structural analog, periplocoside P, has been shown to inhibit V-ATPase activity in the midgut of another lepidopteran, *M. separata* [89].

Our molecular docking analysis (Figure 6) provides a mechanistic hypothesis for these observations. The model suggests that periplocoside D can occupy the binding pocket of *Px*vATPaseE, forming a stable interaction network primarily through hydrogen bonds with conserved threonine and glutamine residues. Based on these results, we predict that periplocoside D acts as a natural insecticide, whose toxicity may be mediated by binding to *Px*vATPaseE. This work, thus, shifts the narrative for periplocoside D from a compound with known toxicity to one with a proposed molecular target, providing a theoretical foundation for the rational design of novel V-ATPase E-targeted insecticides.

## 5. Conclusions

In conclusion, we have identified and validated the V-ATPase subunit E gene as a potent RNAi target for controlling *P. xylostella*, with silencing causing severe developmental defects and mortality. Furthermore, by employing computational virtual screening, we predicted that periplocoside D may bind to the *Px*vATPaseE protein, thereby linking its insecticidal activity to a specific molecular mechanism. These findings present a dual-pronged strategy—genetic (RNAi) and chemical (small-molecule inhibition)—targeting the same essential protein, thereby contributing valuable insights and tools for the development of targeted management strategies against this globally significant pest.:

## Figures and Tables

**Table 1:** Compound name.

Number	CAS Registry Number	Max. Affinity	Compound Name
1	198953-76-3	−9.71	Asperazine
2	120040-21-3	−9.67	Edgeworoside A
3	389122-01-4	−9.42	Karounidiol dibenzoate
4	41059-79-4	−9.195	Timosaponin A-III
5	630057-39-5	−9.162	Neoprzewaquinone A
6	678138-59-5	−9.155	2,3,2”,3”-Tetrahydroochnaflavone
7	36069-05-3	−9.152	Pseudojervine
8	290809-72-2	−9.136	27-Epichantrieroside A
9	57539-70-5	−9.123	Cussonoside A
10	149457-95-4	−9.003	Chaetoglobosin Fex
11	19057-60-4	−8.986	Dioscin
12	7182-54-9	−8.974	Monactin
13	150881-27-9	−8.972	Win 64821
14	46200821	−8.951	CHONGLOU SAPONIN II
15	1422265-57-3	−8.938	Ellagic acid 3-O-alpha-L-rhamnopyranoside
16	131559-54-1	−8.902	Triumbelletin
17	117210-04-5	−8.893	Kaikasaponin I
18	1501943-09-4	−8.881	Trigothysoid O
19	2222584-03-2	−8.862	Triumbelletin 7-O-glucoside
20	135545-89-0	−8.856	Periandradulcin B
21	1800029-50-8	−8.855	Cynanoside F
22	29621-75-8	−8.848	Friedelin 3,4-lactone
23	118555-84-3	−8.831	Floribundone 1
24	60976-49-0	−8.816	Geraniin
25	32854-75-4	−8.81	Lappaconitine
26	1000995-47-0	−8.801	Chloramultilide B
27	114902-16-8	−8.791	Yemuoside YM10
28	94617-36-4	−8.791	Mulberrofuran K
29	1467083-09-5	−8.789	Hybridaphniphylline B
30	55916-51-3	−8.734	Polyphyllin VI
31	116709-64-9	−8.723	Periplocoside D
32	71-63-6	−8.72	Digitoxin
33	438578-91-7	−8.696	Demethyldaphnoretin-7-O-glucoside
34	20347-71-1	−8.693	Procyanidin proanthocyanidins

## Data Availability

The original contributions presented in this study are included in this article/Supplementary Materials. Further inquiries can be directed to the corresponding authors.

## Supplementary Materials

The following supporting information can be downloaded at: https://www.mdpi.com/article/10.3390/insects17040439/s1, Table S1: A list of primers used for RT-PCR of the genes; Figure S1: A display of nucleic acid sequences of *PxvATPaseE* from *Plutella xylostella*; Figure S2: On-target/off-target prediction and high-risk off-target regions of *dsvATPaseE*.
