# Supplementary material for: RNAi-Mediated Silencing of vATPase Subunit E Impairs Larval Development in Plutella xylostella, and Virtual Screening Identifies a Potential Inhibitor
Source: Insects. 2026 Apr 20;17(4):439. doi: 10.3390/insects17040439 (PMC13116757; doi:10.3390/insects17040439)
Supplement: Supplementary file 1 [file insects-17-00439-s001.zip › insects-4162168-supplementary.pdf]

Supplementary Data

RNAi-Mediated Silencing of vATPase Subunit E Impairs Larval Development in *Plutella xylostella*, and Virtual Screening Identifies a Potential Inhibitor

Table S1. Primers used in RT-PCR, dsRNA synthesis and qPCR.

Fragment name	Forward primer	Reverse primer
**RT-PCR**		
PxvATPaseE	ATGGCGCTCAGCGATGCA	TTAGTCGGTGAACTTGCGGTT
**dsRNA synthesis**		
ds*vATPaseE*	GCAAATGAAAAGGCCGAAGA	GGAACAGCGCCTGC
ds*GFP*	TAATACGACTCACTATAGG	CCTATAGTGAGTCGTATTA
**qPCR**		
q*PxvATPaseE*	CAGGCGCAGTACAAGGAGAA	TTAGTCGGTGAACTTGCGGT
q*PxRPL32*	CCAATTTACCGCCCTACC	TACCCTGTTGTCAATACCTCT

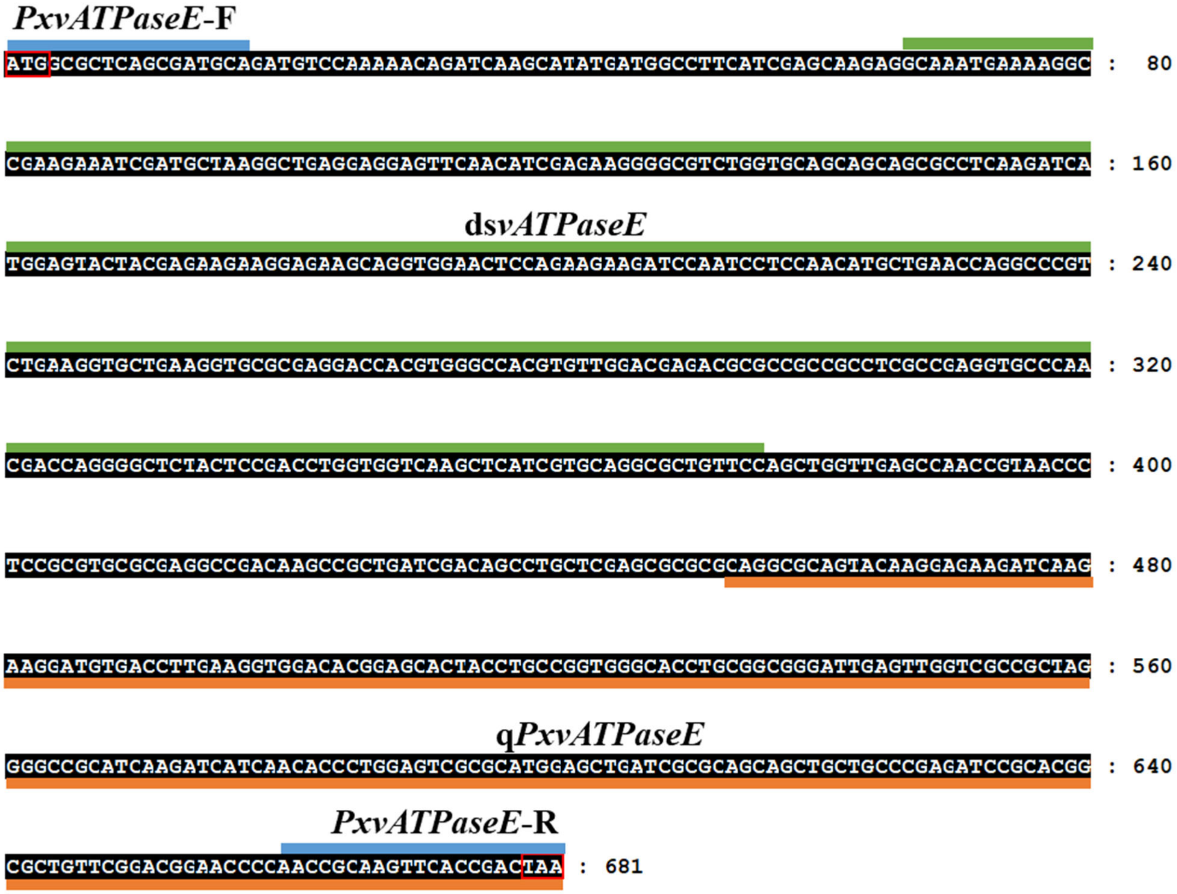

Figure S1. A display of nucleic acid sequences of *PxvATPaseE* from *Plutella xylostella*. Start and stop codons are shown by red boxes. The primers for RT-PCR are indicated with green lines; the sequence for measuring the expression levels of *PovATPaseE* using qRT-PCR is marked with blue line; and the sequence for generation of ds*vATPaseE* is highlighted with red line.

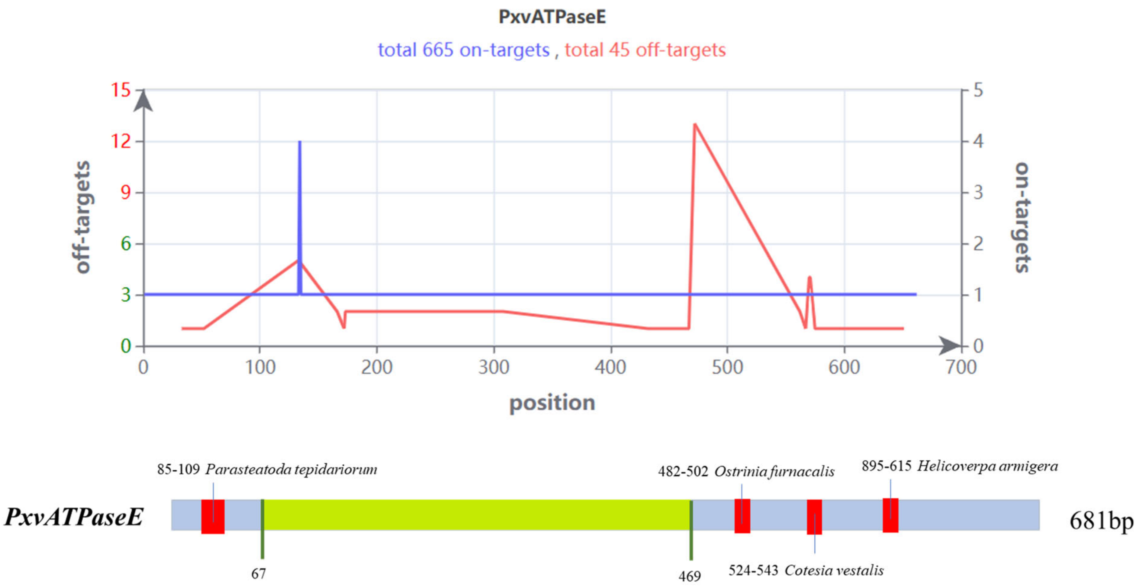

Figure S2. On-target/Off-target Prediction and High-Risk Off-target Regions of *dsvATPaseE*. An online tool (https://www.dsrna-engineer.cn) was employed to predict the quantity and positions of on-target and off-target sites for the *PxvATPaseE* gene fragment during RNAi. The blue curve indicates on-target sites, while the red curve represents off-target sites. Predictions were primarily conducted for arthropod species with predatory relationships or evolutionary similarities to identify potential off-target regions in non-target organisms. The dsRNA was intentionally designed to exclude these high-risk sequence segments, thereby minimizing off-target effects.
